# Imaging cardiac sympathetic innervation with MIBG: linear conversion of the heart-to-mediastinum ratio between different collimators

**DOI:** 10.1186/s40658-019-0250-2

**Published:** 2019-07-23

**Authors:** Joachim Brumberg, Ganna Blazhenets, Nils Schröter, Lars Frings, Wolfgang H. Jost, Constantin Lapa, Philipp T. Meyer

**Affiliations:** 1 Department of Nuclear Medicine, University Hospital Würzburg and Julius-Maximilians-University, Oberdürrbacher Straße 6, 97080 Würzburg, Germany; 2 Department of Nuclear Medicine, Medical Center, Faculty of Medicine, University of Freiburg, Hugstetter Straße 55, 79106 Freiburg im Breisgau, Germany; 3 Department of Neurology, Medical Center, Faculty of Medicine, University of Freiburg, Breisacher Straße 64, 79106 Freiburg im Breisgau, Germany; 4 Center for Geriatrics and Gerontology Freiburg, Medical Center, Faculty of Medicine, University of Freiburg, Lehener Straße 88, 79106 Freiburg im Breisgau, Germany; 5 Parkinson-Klinik Ortenau, Kreuzbergstraße 12, 77709 Wolfach, Germany

**Keywords:** MIBG, Collimator, Heart-to-mediastinum ratio, Linear conversion

## Abstract

**Background:**

The heart-to-mediastinum (H/M) ratio is a commonly used parameter to measure cardiac I-123 metaiodobenzylguanidine (MIBG) uptake. Since the H/M ratio is substantially influenced by the collimator type, we investigated whether an empirical linear conversion of H/M ratios between camera systems with low-energy (LE) and medium-energy (ME) collimator is possible.

**Methods:**

We included 18 patients with parkinsonism who were referred to one of the two participating molecular imaging facilities for the evaluation of cardiac sympathetic innervation by MIBG scintigraphy. Two consecutive planar image datasets were acquired with LE and ME collimators at 4 h after MIBG administration. Linear regression analyses were performed to describe the association between the H/M ratios gained with both collimator settings, and the accuracy of a linear transfer of the H/M ratio between collimators and across centers was assessed using a leave-one-out procedure.

**Results:**

H/M ratios acquired with LE and ME collimators showed a strong linear relationship both within each imaging facility (*R*^2^ = 0.99, *p* < 0.001 and *R*^2^ = 0.90, *p* < 0.001) and across centers (H/M-LE = 0.41 × H/M-ME + 0.63, *R*^2^ = 0.97, *p* < 0.001). A linear conversion of H/M ratios between collimators and across centers was estimated to be very accurate (mean absolute error 0.05 ± 0.04; mean relative absolute error 3.2 ± 2.6%).

**Conclusions:**

The present study demonstrates that a simple linear conversion of H/M ratios acquired with different collimators is possible with high accuracy. This should greatly facilitate the exchange of normative data between settings and pooling of data from different institutions.

## Background

Cardiac imaging with scintigraphy and I-123 metaiodobenzylguanidine (MIBG) is a widely accepted method to assess the integrity and activity of cardiac sympathetic innervation. MIBG is an analog of guanethidine, and its uptake by the postganglionic presynaptic nerve ending is mediated by the noradrenaline uptake and storage mechanisms [1, 2]. Cardiac MIBG uptake in patients with ischemic heart disease and cardiomyopathy has been demonstrated to identify high-risk patients in the context of congestive heart failure [3, 4]. In addition, MIBG imaging is used to identify postganglionic cardiac sympathetic denervation in patients with Lewy body diseases (Parkinson’s disease (PD) and dementia with Lewy bodies (DLB)), which facilitates the differential diagnosis of parkinsonism (most notably, PD vs. multiple system atrophy) [5–7] and of dementia (most notably, DLB vs. Alzheimer’s dementia) [5, 8].

The outcome measure of MIBG scintigraphy is the heart-to-mediastinum (H/M) ratio, which can be estimated using early and, more commonly, late anterior planar images (15 min and 4 h after injection, respectively). Aside from other technical factors (e.g., placement of regions of interest, ROI), the H/M ratio is known to be strongly influenced by the use of distinct camera-collimator configurations. This hampers the transfer of normative data across institutions and pooling of data in multi-center studies. In particular, collimator choice has a substantial effect on the semi-quantitative assessment of cardiac MIBG uptake: scatter and septal penetration of radiation emitted by the heart and surrounding organs (especially the liver) influence planar H/M ratios [9]. The strongest error due to septal penetration has been reported for low-energy high-resolution (LE) collimators [9, 10], while medium-energy (ME) collimators can minimize these effects [11]. However, due to their greater availability and probably also pragmatic reasons (lack of need to change the collimator from most routine examinations), LE collimators are also commonly used, impeding data transfer between institutions. Previous studies reported a linear relationship between LE and ME collimators based on patient examinations [10, 12] and phantom studies [13, 14]. For an ongoing bi-center study on the diagnostic performance of MIBG scintigraphy in comparison to F-18 fluorodeoxyglucose and positron emission tomography in neurodegenerative parkinsonism, we sought to extend few earlier findings by providing additional data on these particular patient population who were scanned on current SPECT/CT systems. Such a comparison will be of great interest for future multi-center studies including the data from various institutions and camera settings in the fields of cardiology (e.g., ischemic heart disease, cardiomyopathy, congestive heart failure) and neurology (e.g., parkinsonism, dementia). In addition to earlier studies, we also used a leave-one-out procedure for proper validation of the empiric conversion in a sample with parkinsonism. Moreover, we hypothesized that a relatively small number of MIBG scans (≤ 10) employing both LE and ME collimators suffices to reliably describe the relationship and allows for an empiric conversion.

## Methods

### Patients

Eighteen consecutive patients with suspected neurodegenerative parkinsonism (i.e., mostly differential diagnosis of PD vs. multiple system atrophy; 14 males; 66.4 ± 8.2 years), which were referred for cardiac MIBG scintigraphy to the Departments of Nuclear Medicine of the University Hospital Freiburg (center a; 6 males, 4 females; 63.6 ± 7.3 years) and University Hospital Würzburg (center b; 8 males; 70.0 ± 8.2 years) were enrolled in this study.

### MIBG scintigraphy

Patient preparation was implemented according to the guidelines of the European Association of Nuclear Medicine [11, 15]. Any drugs with known interference with MIBG uptake (e.g., tricyclic antidepressants) were discontinued for a minimum withdrawal time recommended by the guidelines [11, 15] or (if not specified) for at least 5 plasma half-lives. Potassium perchlorate was used to prevent thyroid uptake of free iodine. MIBG studies were acquired on two dual-headed SPECT/CT systems: Brightview XCT (center a; Philips Medical Systems Inc., Cleveland, OH) and Symbia T2 (center b; Siemens Healthineers, Erlangen, Germany). Brightview XCT was equipped with a 3/8-inch crystal and scans were conducted with LE and ME general-purpose collimators (photopeak window of 159 keV ± 20%). Acquisitions on Symbia T2 (5/8 in. crystal) were performed using LE and ME low penetration collimators (photopeak window of 159 keV ± 15%). Anterior and posterior planar images were obtained twice for 5 min at 4 h after the injection of 183 ± 25 MBq MIBG. The first acquisition was performed with LE, subsequently, the scan was repeated with ME collimator. Scintigrams of one patient were performed in the reversed order. Of note, we did not employ early MIBG scan (i.e., at 15 min after injection) which is inferior to the late MIBG scan according to an earlier meta-analysis [6]. Imaging was performed as part of the clinical work-up. All patients gave written informed consent prior to the investigations for receiving the respective imaging procedures.

### Image analysis

MIBG uptake was semi-quantitatively evaluated on the planar anterior images by calculating the H/M ratio using the PMOD image analysis software version 3.7 (PMOD Technologies Ltd, Zurich, Switzerland). First, ME images were automatically co-registered to the LE images and fused. Then, two investigators (JB; CL) defined ROIs of the heart and the mediastinum independently from each other on the fused image data. The center of a circle and one rectangular ROI was manually placed on the heart and the upper mediastinum, respectively. The ROIs were then transferred to the co-registered LE and ME image data and H/M ratios were calculated. Finally, the mean H/M ratio of both raters was calculated for each collimator and each patient by dividing the mean counts per pixel in the cardiac ROI by the mean counts per pixel in the mediastinal ROI.

### Statistical analysis

Statistical analysis was performed with the commercial software package SPSS 25.0 (IBM Corp., Armonk, NY, USA). We explored inter-rater agreement of H/M ratio with the intra-class correlation coefficient (ICC) [16]. Linear regression analyses were employed to describe the association between H/M ratios acquired with LE and ME collimators for both centers individually and pooled together. Analysis of covariance (ANCOVA) was used to test the interaction between center and collimator to evaluate if regression slopes depend upon study center. Finally, we performed a leave-one-out cross-validation (LOOCV) to test the validity of an empiric linear transfer of H/M ratios from ME to LE collimators with an in-house pipeline in MATLAB and Statistics Toolbox Release R2017a (The MathWorks, Inc., Natick, MA, USA). The deviation of the H/M ratio predicted by linear conversion from the actually measured H/M ratio was assessed by calculating the absolute difference (absolute error) and relative absolute difference (relative absolute error; expressed as percentage relative to the measured value).

## Results

**Fig. 1:**
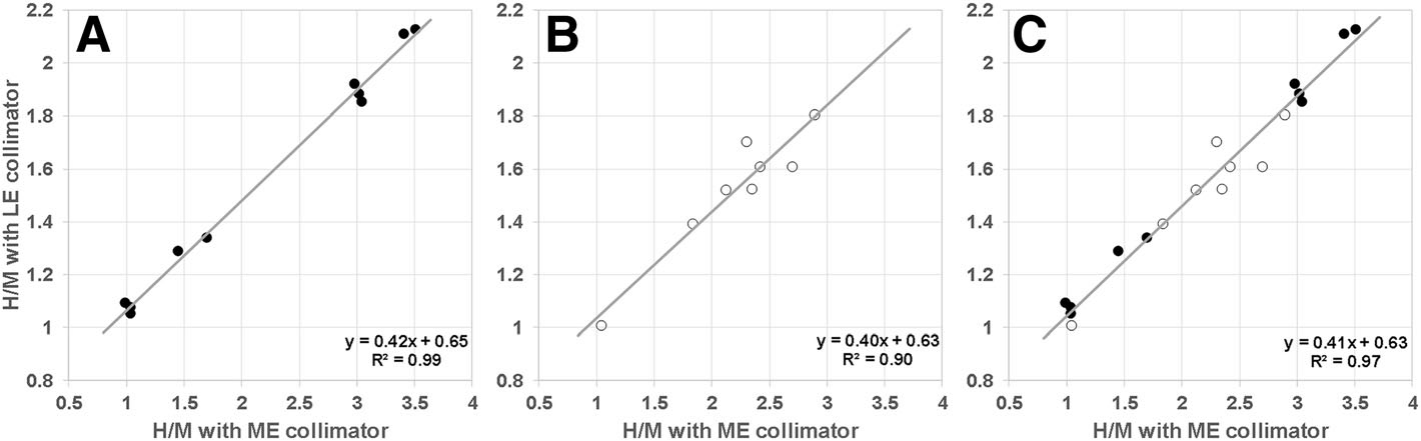
Scatter plots and linear regression analyses between the MIBG heart-to-mediastinum ratio (H/M) acquired with low-energy collimators (LE) in comparison to the H/M ratio obtained with medium-energy collimators (ME). Plots are given separately for each center (**a**, Freiburg; **b**, Würzburg) and pooled together (**c**). Closed circles indicate subjects from center **a**, and open circles indicate those from center **b**

Inter-rater assessment revealed a very high agreement of H/M ratios between both raters (ICC = 0.99). Image acquisition using ME collimators resulted in higher H/M values (mean ± standard deviation 2.22 ± 0.86; range 1.00–3.51) as compared to LE collimators (1.55 ± 0.36; 1.01–2.13). H/M ratio acquired with LE collimators exhibited highly significant linear correlations with the corresponding ratio obtained with ME collimators, both when contemplating each center separately (center a: H/M-LE = 0.42 × H/M-ME + 0.65, *R*^2^ = 0.99, *p* < 0.001; Fig. 1a; center b: H/M-LE = 0.40 × H/M-ME + 0.63, *R*^2^ = 0.90, *p* = 0.003; Fig. 1b) and when pooling the data of both centers together (H/M-LE = 0.41 × H/M-ME + 0.63, *R*^2^ = 0.97, *p* < 0.001; Fig. 1c). ANCOVA revealed homogeneity of regression slopes (*p* = 0.757). Using LOOCV, we found very similar to identical regression parameters both within (center a: H/M-LE = 0.42 × H/M-ME + 0.65, *R*^2^ = 0.99; center b: H/M-LE = 0.40 × H/M-ME + 0.65, *R*^2^ = 0.89) and across centers (H/M-LE = 0.41 × H/M-ME + 0.63, *R*^2^ = 0.97), underlining the robustness of the association.

Based on LOOCV, the mean (±SD) absolute error of empiric linear conversion was 0.08 ± 0.06 (center b) and 0.04 ± 0.02 (center a) when contemplating both centers separately, and 0.05 ± 0.04 when pooling the data. The mean relative absolute error was only 2.4% ± 1.2% when considering only data of center a and slightly larger (5.4% ± 5.1%) when considering only data of center b. Finally, mean relative absolute error was only 3.2% ± 2.7% (never exceeding 10% in individual cases) when pooling the data of both centers. Interestingly, when applying the regression equation of one center for conversion of the data of the other center (and vice versa), the conversion errors (e.g., relative absolute error: center a, 3.0% ± 2.0%; center b, 4.7% ± 3.2%) were comparable to those gained by LOOCV, underlining that the effect between LE and ME collimators is much stronger than the effect of different camera systems.

## Discussion

This study investigated the association of late MIBG H/M ratios successively acquired with LE and ME collimators and in how far an empirical linear conversion of H/M ratios is accurately possible between different collimators. It extends a few previous studies (in part only phantom studies) by providing additional data on parkinsonism patients that was acquired on state-of-the-art SPECT/CT systems and analyzed by a proper leave-one-out procedure. The observed relationship between H/M ratios was linear in both study centers and highly congruent between centers. These findings confirm previous studies, which described a linear relationship of H/M ratios with similar regression equations (H/M-LE = 0.43 × H/M-ME + 0.64 and H/M-LE = 0.47 × H/M-ME + 0.62) in large cohorts (*n* ≥ 40) using successive imaging with LE and ME collimators [10, 13], and validated the conversion equation by applying it to an independent patient collective [12].

Our results extend these studies by illustrating that a limited number of patients (*n* ≤ 10) undergoing successive acquisitions with both collimators represents a very simple and sufficiently accurate empiric approach to convert H/M ratios between ME and LE collimators. Furthermore, the linear association between both collimator types was found to be independent of the two camera systems enrolled. For instance, and in line with an earlier study [12], our study shows that possible effects of crystal thickness and energy window widths are actually negligible.

These results greatly increase the comparability between camera-collimator configurations and centers. By using LOOCV, we demonstrated that the mean *absolute* error of the H/M ratio was only 0.05 ± 0.04 or, in relative terms, 3.2% ± 2.7% and never exceeded 10% in individual cases. This also compares favorably to earlier studies: Inoue et al. [10, 12] reported a mean error of 0.00 ± 0.18 and 0.00 ± 0.27 for the conversion of LE to ME by simple linear regression (not LOOCV). Using the same approach and direction of conversion, the respective mean ± SD error with our data would be 0.00 ± 0.14 (note, that with this approach the mean error cancels out to zero, while the SD reflect the variation of the error). The higher accuracy can probably be attributed to the use of a fully automatic and highly accurate co-registration of LE and ME datasets so that both datasets could be analyzed using an identical ROI set. Furthermore, it is intriguing to note that the correlation coefficient between both collimators was higher for the data of center a than the data of center b (*R*^2^ = 0.99 vs. 0.90). This is probably due to the fact that the patients in center a did not change their position during collimator change while the patients in center b stood up from the camera bed. Thus, the higher uncertainty of the data from center b could be related to the change of position of the patient and organs after collimator switch. Indeed, a mean test-retest difference for MIBG H/M ratios acquired with LE collimators between 0.06 and 0.08 has been described before [17]. Thus, these two factors should be considered when establishing institutional conversion equations (i.e., use of accurate image co-registration and a unified ROI set; identical body positions).

## Conclusions

The present study demonstrates that a simple linear conversion of H/M ratios acquired with different collimators is possible with high accuracy. The empiric conversion equation can be gained from a very limited number (*n* ≤ 10) of patients undergoing successive imaging with both collimators. This should greatly facilitate the exchange of normative data between settings and pooling of data from different institutions (e.g., in multi-center studies).

## Data Availability

Please contact the corresponding author for data requests.

## Abbreviations

ANCOVA: Analysis of co-variance
DLB: Dementia with Lewy bodies
H/M: Heart-to-mediastinum
ICC: Intra-class correlation coefficient
LE: Low-energy high-resolution
LOOCV: Leave-one-out cross-validation.
ME: Medium-energy
MIBG: Metaiodobenzylguanidine
PD: Parkinson’s disease
ROI: Regions of interest
